# Multidrug-Resistant ESBL-Producing *E. coli* in Clinical Samples from the UK

**DOI:** 10.3390/antibiotics12010169

**Published:** 2023-01-13

**Authors:** Delveen R. Ibrahim, Christine E. R. Dodd, Dov J. Stekel, Remilekun T. Meshioye, Mathew Diggle, Michelle Lister, Jon L. Hobman

**Affiliations:** 1 Department of Biology, School of Science, The University of Duhok, Duhok 42001, Iraq; 2 School of Biosciences, University of Nottingham, Loughborough, Leicestershire LE12 5RD, UK; 3 Department of Mathematics and Applied Mathematics, University of Johannesburg, Rossmore 2029, South Africa; 4 Fidson Healthcare Plc, 268 Ikorodu-Ososun Rd, Obanikoro, Lagos 100232, Nigeria; 5 Alberta Health Services, Edmonton, AB T6G 2J2, Canada; 6 Department of Microbiology, Nottingham University Hospitals NHS Trust, Derby Road, Nottingham NG7 2UH, UK

**Keywords:** *Escherichia coli*, multidrug-resistant, cephalosporin-resistant, ESBL

## Abstract

Globally, cephalosporin therapy failure is a serious problem for infection control. One causative agent of cephalosporin-resistant infections is multidrug-resistant (MDR) *E. coli* producing extended-spectrum β-lactamases (ESBLs) and/or plasmid-encoded AmpC (pAmpC) β-lactamases. We evaluated the occurrence of ESBL/pAmpC genetic determinants in phenotypically MDR *E. coli* isolated from clinical samples of blood, faeces, ear effusion, urine and sputum from a UK hospital. Phenotypic resistance profiling for 18 antibiotics (from seven classes) showed that 32/35 isolates were MDR, with resistance to 4–16 of the tested antibiotics. Of the isolates, 97.1% showed resistance to ampicillin, 71.4% showed resistance to co-amoxiclav, cefotaxime, ceftazidime and ceftiofur, and 68.5% showed resistance to cefquinome. *bla*_CTX-M_, *bla*_TEM_ and *bla*_OXA-1_ genes were detected in 23, 13 and 12 strains, respectively, and *Intl1* was detected in 17 isolates. The most common subtypes among the definite sequence types were CTX-M-15 (40%) and TEM-1 (75%). No *E. coli* isolates carried pAmpC genes. Significant correlations were seen between CTX-M carriage and cefotaxime, ceftiofur, aztreonam, ceftazidime and cefquinome resistance; between *bla*_CTX-M_, *bla*_TEM_ and *bla*_OXA-1_ carriage and ciprofloxacin resistance; and between *Intl1* carriage and trimethoprim/sulfamethoxazole resistance. Thus, MDR phenotypes may be conferred by a relatively small number of genes. The level and pattern of antibiotic resistance highlight the need for better antibiotic therapy guidelines, including reduced use and improved surveillance.

## 1. Introduction

It has been estimated that, in total, about 700,000 people die every year from drug-resistant strains of HIV, malaria, multidrug-resistant and extremely drug-resistant tuberculosis (TB) and common bacterial infections. It has been predicted that this might reach 10 million cases by 2050 unless action is taken [1,2]. In 2019, approximately 929 000 deaths worldwide were found to be attributable to six leading pathogens that are associated with antimicrobial resistance (*Escherichia coli*, *Staphylococcus aureus*, *Klebsiella pneumoniae*, *Streptococcus pneumoniae*, *Acinetobacter baumannii* and *Pseudomonas aeruginosa*) [3]. In the UK, it has been reported that the estimated number of deaths attributable to antibiotic-resistant bacteria increased year by year from 2016 and 2019, with a small decline in 2020 (estimated 2596 deaths in 2019 versus 2228 in 2020) [4]. Extended-spectrum β-lactamase (ESBL)-producing *Escherichia coli* and other Enterobacteriaceae are a major cause of resistance to expanded-spectrum β-lactam antibiotics. Since the discovery of ESBL in the early 1980s [5,6], ESBL genes have spread worldwide and are now endemic in Enterobacteriaceae, which are isolated from both hospital-associated and community-acquired infections [7], livestock and environmental samples [8,9,10,11]. It has been estimated that ESBL-producing *E. coli* caused more than 5000 cases of bacteraemia annually in the UK [12]. In an epidemiological surveillance and typing study performed in the UK, it was found that the original source of 60% of ESBL-producing *E. coli* that caused bacteraemia was the genitourinary tract [12]. In the UK, AMR was common in more than 1 million UTIs caused by bacteria identified in NHS laboratories in 2016 (PHE, 2017). Currently, *E. coli* is considered the most common bloodstream pathogen in England, with an increase in the case count from 32,309 in the financial year 2012–2013 to 43,368 in 2019–2020. In Nottingham and Nottinghamshire, the cases increased from 772 cases in 2012–2013 to 925 in 2019–2020 [13].

In general, *E. coli* is considered a commensal of the gastrointestinal tract. However, random point mutations or the acquisition of virulence factors, both chromosomal and extrachromosomal, can make this commensal bacterium become an extremely adapted pathogen [14,15]. There are two main groups of disease-causing *E. coli*: the first causes diarrhoeal diseases and is often described as intestinal pathogenic *E. coli* (IPEC), while the second group causes infections outside of the gut and is called extraintestinal pathogenic *E. coli* (ExPEC) [16,17]. The latter can cause a variety of serious infections, such as urinary tract infections, septicaemia and meningitis [18], which require antibiotic treatment. It is in these species that the emergence of multidrug resistance is a major cause for concern [14,19].

Beta-lactam antibiotics, including third-generation cephalosporins, are the most frequently used antibiotics worldwide. Consequently, bacterial resistance has continued to rise since their introduction due to a constant selective influence for resistance to them [6,20]. β-Lactamases, which are naturally occurring in some species of bacteria, have become mobilised by transposable elements and have become widespread in response to the use and overuse of β-lactam antibiotics [7]. In the early 1970s, resistance to ampicillin and penicillins in combination with β-lactamase inhibitors, for example, amoxicillin and clavulanic acid, emerged due to plasmid-encoded β-lactamases [21]. This resulted in the increased use of alternatives, especially cephalosporins [22].

The carriage of β-lactamase and ESBL genes on some transposable elements has been reported previously [23,24,25]. Plasmids and transposons that carry AMR genes can also carry genes facilitating resistance to toxic metals; for example, Tn*21* encodes resistance to mercuric ions, streptomycin and sulphonamides [26,27,28]. This can contribute to the generation of multiresistant phenotypes.

Extended-spectrum cephalosporinases belong to group 2 β-lactamases according to the Bush and Jacoby classification [29]; this group includes penicillinases, early cephalosporinases and extended-spectrum cephalosporinases and serine carbapenems, of which most are inactivated by β-lactamase inhibitors, such as clavulanic acid. Extended-spectrum cephalosporinases work on different substrates, such as substrates in the broad-spectrum group (for example, penicillin and cefazolin), as well as oxyimino-cephalosporins (cefotaxime, cefpodoxime, ceftazidime and ceftriaxone), monobactam (aztreonam) and, for some enzymes, cefepime as well [6,29].

ESBLs are serine β-lactamases, belonging to class A of the Ambler molecular and structural classification and to Group 2be according to the Bush and Jacoby classification [6,29,30,31]. ESBLs are biochemically characterised by their ability to hydrolyse expanded-spectrum β-lactam antibiotics and their inhibition by β-lactamase inhibitors, specifically clavulanate; they can confer resistance to fourth-generation cephalosporins but cannot hydrolyse cephamycins. AmpC belongs to class C of the Ambler classification and Group 1 according to the Bush, Jacoby and Mederios classification; biochemically, they are poorly inhibited by clavulanic acid and cannot hydrolyse fourth-generation cephalosporins but can hydrolyse cephamycins [6,29,32,33,34].

The surveillance of AMR pathogens is critical to understanding the local situation and the distribution of important pathogens that have the ability to cause various infections, including bacteraemia, urinary tract infections and intestinal infections. Detecting the level of AMR for the causative agent of the infection is very important and has a significant role in treatment choices. The aim of this study was to investigate the mechanisms of β-lactam resistance present in cephalosporin-resistant *E. coli* from different clinical isolates; to detect the different ESBL genes and their variant types; to detect the phenotypic resistance pattern of those isolates against 18 different antibiotics; and to determine whether specific resistance patterns were associated with ESBL resistance, particularly the presence of *Intl1, merA* and *merC* genes.

## 2. Results

### 2.1. Strains

All isolates were putatively identified as *E. coli* in the clinical samples, but further confirmation was performed to check their identity. Of the 35 isolates, 21 were isolated from blood (B), 8 were isolated from urine (U), 2 were isolated from sputum (R), 3 were isolated from faeces (F) and 1 was isolated from an ear infection (E). All isolates except two showed a blue/green colour on TBX agar and were indole-positive and oxidase-negative, which further confirmed their identification as *E. coli*. The other two isolates (H10B and H20U) grew as white colonies on TBX agar and gave negative indole and negative oxidase test results. 16S rDNA sequencing was used to check the identity of one of them (H10B), and the sequence types confirmed these isolates as *Klebsiella pneumoniae*.

### 2.2. Antibiotic Resistance

**Figure 1:**
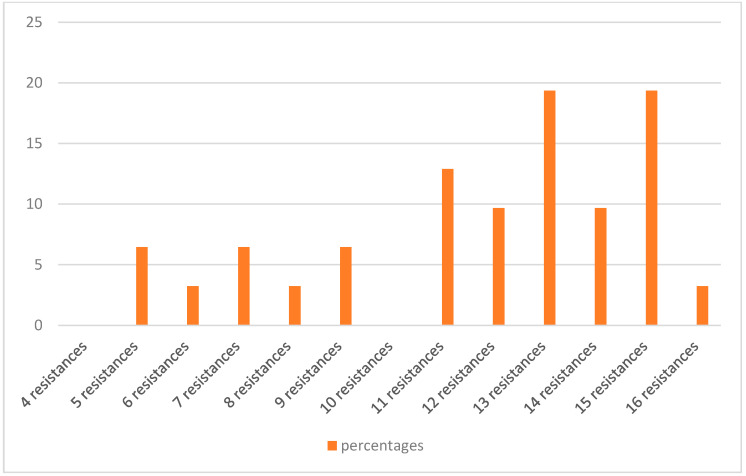
The percentages of MDR *E. coli* resistant to different numbers of antibiotics.

**Figure 2:**
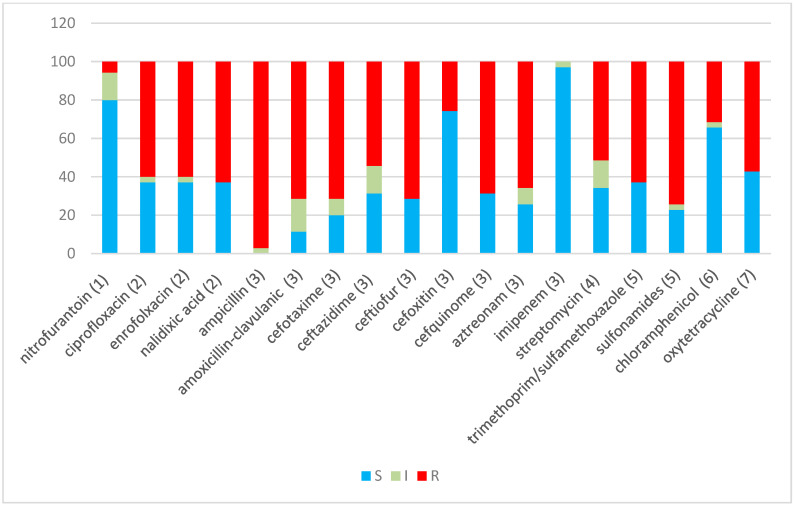
Percentage sensitivity to 18 antibiotics for 35 *E. coli* and non-*E. coli* isolates. Blue indicates sensitive, grey indicates intermediate sensitivity and red indicates resistance percentages, using CLSI (2013) definitions. The Y-axis represents the percentage of isolates. Antibiotic classes: (1) nitrofuran derivative, (2) quinolones, (3) β-lactams, (4) aminoglycoside, (5) sulphonamide/complex, (6) phenicol and (7) tetracycline.

Of the isolates, 91.4% (32 isolates) were found to be MDR, as they showed resistance to at least three different classes of antibiotics [35]. Only one isolate (H29F) showed intermediate resistance to a single antibiotic, while another two (H33F and H6B) showed resistance or intermediate resistance to 4–5 antibiotics, but all belong to one class of antibiotic, which does not comply with the definition of MDR (Table 1). One isolate (H12B) showed resistance or intermediate resistance to 16 antibiotics, while another five showed resistance or intermediate resistance to 15 antibiotics (Figure 1). Figure 2 shows the percentages of strains resistant to each antibiotic. The highest percentage of phenotypic resistance amongst the *E. coli* strains was to ampicillin (97.1%), followed by 74.2% for sulphonamide and 71.4% for amoxicillin–clavulanic acid, cefotaxime and ceftiofur, while 25.7% showed resistance to cefoxitin. Cefquinome, a member of fourth-generation cephalosporins, and aztreonam, one of the monobactams, showed no effectiveness against 68.5% and 65.7% of the isolates, respectively. Quinolone’s antibiotic effectiveness was poor, as 62.8% of the isolates were resistant to nalidixic acid, and 60% of the isolates were resistant to ciprofloxacin and enrofloxacin. The aminoglycoside streptomycin showed no effect against 51.4% of the isolates, and 62.8% of the isolates showed resistance to trimethoprim (a dihydrofolate reductase inhibitor) in combination with sulfamethoxazole, while 31% and 57% of the isolates were resistant to chloramphenicol and oxytetracycline. No *E. coli* isolates showed resistance to imipenem. A total of 17.1% of isolates exhibited intermediate resistance to amoxicillin–clavulanic acid, while 14.2% exhibited intermediate resistance to nitrofurantoin, streptomycin and ceftazidime.

**Table 1:** Isolates with β-lactamase genes, *Intl1*, *merA* and *merC*, the phenotypic results of ESBL/AmpC screening and their phenotypic resistance patterns for different antibiotics.

Isolate ID	β-Lactamases	Gene Sequence Type	*Intl1*/*merA* and *merC*	ESBL Screening Test	AmpC Screening Test	Antimicrobial Resistance Profile
H1B	CTX-M/OXA	CTX-M-55/OXA-1	*Intl1*	+ *		* STX, AMC, OT, CAZ, NA, EFT, S300, CFQ, ATM, AMP, ENR, CTX, CIP
H2B	CTX-M/OXA	CTX-M-285/OXA-1	*Intl1*	+		STX, AMC, CAZ, NA, EFT, S300, CFQ, ATM, AMP, ENR, CTX, CIP
H3B	CTX-M/OXA	CTX-M-15/OXA-1		+		AMC, OT, CAZ, NA, EFT, CFQ, ATM, AMP, (C), ENR, CTX, CIP
H4B	CTX-M/TEM	ND/TEM-1		+	-	AMC, OT, S10, CAZ, NA, EFT, S300, CFQ, ATM, AMP, C, ENR, CTX, CIP, FOX
H5B	CTX-M/TEM/	CTX-M-15/ TEM-1		+		AMC, OT, S10, CAZ, NA, EFT, S300, CFQ, ATM, AMP, C, ENR, CTX, CIP
H6B	TEM			−	-	AMC, (ATM), AMP, (CTX), FOX
H7B	CTX-M/OXA	ND/OXA-1	*Intl1*	+		STX, OT, (S10), (CAZ), NA, EFT, S300, CFQ, ATM, AMP, ENR, CTX, CIP
H8B	CTX-M/OXA	CTX-M-254/OXA-1	*Intl1*	+		STX, (AMC), OT, CAZ, NA, EFT, S300, CFQ, ATM, AMP, ENR, CTX, CIP
H9B	CTX-M/OXA	ND/OXA-1	*Intl1*	+		STX, AMC, OT, (S10), (CAZ), NA, EFT, S300, CFQ, ATM, AMP, ENR, CTX, CIP
H10B	CTX-M/SHV/TEM/OXA/plasmid AmpC	ND/ND/ND/OXA-1/CIT/FOX	*Intl1*	ND	ND	STX, AMC, OT, S10, EFT, S300, CFQ, ATM, AMP, CTX, (CIP)
H11B	CTX-M/TEM	ND/ND		+	-	AMC, OT, S10, CAZ, NA, EFT, S300, CFQ, ATM, AMP, C, ENR, CTX, CIP, FOX
H12B	CTX-M	ND		+	-	AMC, (F), OT, S10, CAZ, NA, EFT, S300, CFQ, ATM, AMP, C, ENR, CTX, CIP, FOX
H13B	CTX-M	ND		+		STX, S10, OT, CAZ, NA, EFT, S300, CFQ, ATM, AMP, C, ENR, CTX, CIP
H14B	CTX-M	ND	*Intl1*	+		STX, (S10), CAZ, NA, EFT, S300, CFQ, ATM, AMP, C, ENR, CTX, CIP
H15B	CTX-M/OXA	CTX-M-15/OXA-1	*Intl1*	+		STX, AMC, (S10), CAZ, NA, EFT, S300, CFQ, ATM, AMP, C, CTX, CIP
H16B	CTX-M	ND	*Intl1*	+		STX, AMC, CAZ, NA, EFT, S300, CFQ, ATM, AMP, ENR, CTX, CIP
H17B				+	-	AMC, OT, S10, CAZ, NA, EFT, S300, CFQ, ATM, AMP, C, ENR, CTX, CIP, FOX
H18B				+	-	AMC, OT, S10, CAZ, NA, EFT, S300, CFQ, ATM, AMP, C, ENR, CTX, CIP, FOX
H19U			*Intl1*	−	-	STX, AMC, (F), OT, S10, (CAZ), NA, S300, AMP, ENR, (CTX), CIP, FOX
H20U	SHV/TEM/plasmid AmpC	ND/TEM-1/CIT/FOX	*Intl1*/*merA*	ND	ND	SXT, AMC, F, OT, (IMP), S10, S300, AMP
H21U	CTX-M/TEM	ND/ND	*merA*/*merC*	+		STX, AMC, S10, (CAZ), EFT, S300, CFQ, (ATM), AMP, ENR, CTX
H22U	CTX-M/TEM	CTX-M-14/TEM-1		+		STX, (AMC), S10, EFT, S300, CFQ, (ATM), AMP, CTX
H23U	TEM		*Intl1*	ND		STX, AMC, NA, S300, AMP, (ENR)
H24U	TEM	TEM-30		ND		STX, AMC, S10, AMP
H25R	TEM	TEM-32	*Intl1*	ND		AMC, F, OT, S10, AMP
H26B	CTX-M/TEM	CTX-M-15/TEM-1	*Intl1*/*merA*/ *merC*	+	-	STX, AMC, S10, CAZ, EFT, S300, CFQ, ATM, AMP, C, CTX
H27B	CTX-M	CTX-M-55		+		AMC, CAZ, EFT, ATM, AMP, CTX
H28E	CTX-M/OXA	ND/OXA-1	*Intl1*	+	-	STX, AMC, OT, CAZ, NA, EFT, S300, CFQ, ATM, AMP, C, ENR, CTX, CIP, FOX
H29F				−		(AMP)
H30R	CTX-M/OXA	ND/OXA-1		+		AMC, OT, CAZ, NA, EFT, CFQ, ATM, AMP, ENR, CTX, CIP
H31B	CTX-M/OXA	ND/OXA-1	*Intl1*	+		STX, AMC, (S10), CAZ, NA, EFT, S300, CFQ, ATM, AMP, ENR, CTX, CIP
H32F	CTX-M/OXA	CTX-M-254/OXA-1	*Intl1*	+		STX, AMC, (F), OT, S10, CAZ, NA, EFT, S300, CFQ, ATM, AMP, ENR, CTX, CIP
H33F	TEM	TEM-1		−	+	AMC, AMP, (CTX), FOX
H34U				ND	ND	STX, AMC, (F), OT, S10, S300, AMP
H35U				ND	ND	STX, AMC, (F), OT, S10, S300, AMP

+ Means positive for the test; − means negative for the test; * for the antibiotic abbreviations, see Table 2; the isolates in red typeface belong to cluster 1 in the ERIC dendrogram.

**Table 2:** Antimicrobial assay discs, abbreviations, classes and amount of antibiotic contained in each disc.

Antibiotics/Abbreviation	Antimicrobial Class	Disc Content
Ampicillin (AMP)	Β-Lactam/Penicillin	10 μg
Amoxicillin–clavulanic acid (AMC)	Β-Lactam/Penicillin Combination with Beta-Lactamase Inhibitor	20/10 μg
Aztreonam (ATM)	Β-Lactam/Monobactam	30 μg
Cefotaxime (CTX)	Β-Lactam/Third-Generation Cephalosporin	30 μg
Ceftazidime (CAZ)	Β-Lactam/Third-Generation Cephalosporin	30 μg
Cefquinome (CFQ)	β-Lactam/Fourth-Generation Cephalosporin	30 μg
Ceftiofur (EFT)	β-Lactam/Third-Generation Cephalosporin	30 μg
Cefoxitin (FOX)	β-Lactam/Second-Generation Cephalosporin	30 μg
Imipenem (IMP)	β-Lactam/Carbapenem	10 μg
Streptomycin (S10)	Aminoglycoside	10μg
Ciprofloxacin (CIP)	Quinolone/Fluoroquinolone	5 μg
Enrofloxacin (ENR)	Quinolone/Fluoroquinolone	5 μg
Nalidixic acid (NA)	Quinolone	30 μg
Nitrofurantoin (F)	Nitrofuran derivative	300 μg
Chloramphenicol (C)	Phenicol	30 μg
Trimethoprim–sulfamethoxazole (SXT)	Sulphonamide/complex	1.25/23.75 μg
Sulphonamide (S300)	Sulphonamide	300 μg
Oxytetracycline (OT)	Tetracycline	30 μg

### 2.3. Phenotyping of ESBL/AmpC and PCR Typing of ESBL Genes

ESBL phenotypes were tested for all 27 of the isolates that showed resistance or intermediate resistance to one of the cephalosporins (CTX, CAZ or ceftiofur) in the initial antibiotic sensitivity assays. The test showed that 24/27 (88.8%) isolates were phenotypically ESBL using confirmatory test kits. The AmpC phenotype test was performed for all nine isolates that showed resistance to cefoxitin in the initial antibiotic sensitivity tests. However, cefoxitin resistance can also be due to reduced cell wall permeability [36], so full confirmation was performed to determine whether the inhibition zone diameter for CTX + cloxacillin and CAZ + cloxacillin discs was ≥5 mm larger than those for the discs containing CTX and CAZ antibiotics alone, as AmpC activity against cephalosporins is inhibited by cloxacillin. Only one isolate (H33F) gave positive results in the test (Table 1); the other eight cefoxitin-resistant isolates might therefore be due to temporary chromosomal AmpC overproduction.

The isolates that showed resistance or intermediate resistance to one of the cephalosporins (CTX, CAZ or EFT) were also tested by PCR for the presence/absence of *bla*_CTX-M_, *bla*_SHV_, *bla*_TEM_, *bla*_OXA-1_ and *bla*_OXA-2_ β-lactamases. *bla*_CTX-M_ was detected in 23 isolates (65.7%), and *bla*_TEM_ was detected in 13 isolates (37.1%), of which 5 carried both genes, for example, H5B and H26B. *bla*_OXA-1_ was present in 12 isolates (34.2%); interestingly all of the isolates that carried *bla*_OXA-1_ carried *bla*_CTX-M_ as well, for example, H1B and H2B. Some of the positive CTX-M and TEM isolates were sequenced, and the results were submitted to NCBI databases. CTX-M-15 (4/10 isolates) and TEM-1 (6/8 isolates) were the most common subtypes among the definite sequences (Table 1. With regard to *bla*_SHV_ and pAmpC, only two isolates showed positive results, but they were not *E. coli* isolates (H10B and H20U); the pAmpC variants belonged to the CIT and FOX families.

### 2.4. Intl1, merA and merC Detection

The presence of *Intl1*, *merA* and *merC* genes was used as an indicator of the presence of a class 1 integron, located within a Tn*21*- or Tn*21*-like mercury resistance transposon. Through PCR analysis, 17 isolates were found to be carrying *Intl1*, with 8 of these found in combination with *bla*_CTX-M_ and *bla*_OXA-1_, for example, H7B and H8B. *merA* was detected in three isolates and *merC* was found in two isolates, with only one isolate (H26B) found to carry the three genes together, along with other genes (*bla*_CTX-M_ and *bla*_TEM_). Another isolate (H21U) carried the *merA* and *merC* genes in combination with *bla*_CTX-M_ and *bla*_TEM_ (Table 1).

### 2.5. Cluster Analysis of the Antibiotic Sensitivity Results with Different Genes and Screening Tests

**Figure 3:**
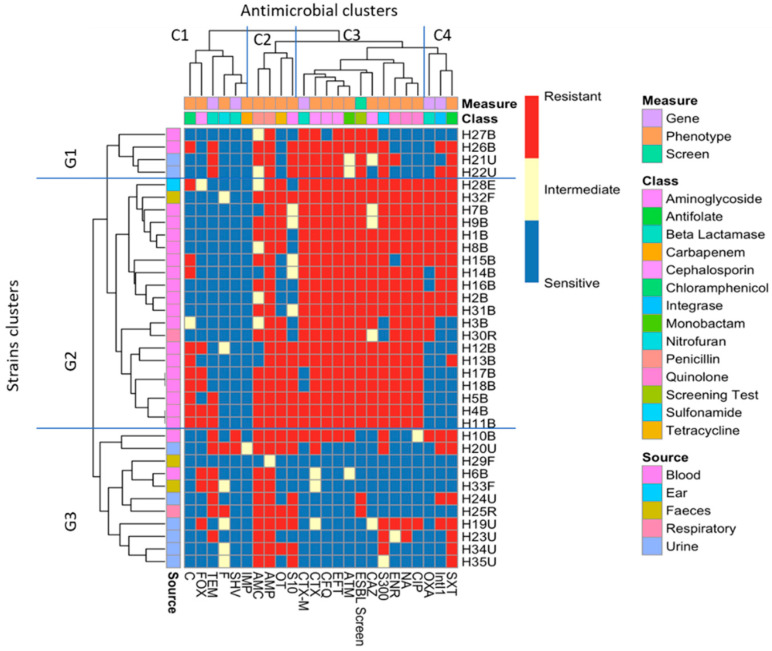
Heatmap of bacterial strains clustered according to both phenotype and gene carriage. G1, G2 and G3 represent the clustering of the isolates according to their antibiotic resistance and gene carriage profiles, while the top of the heatmap (C1, C2, C3 and C4) represents the clustering of the antibiotics, β-lactamase and ESBL genes, ESBL phenotypic test and the *Intl1*.

Cluster analysis of the isolates (Figure 3, vertical axis) showed that, with some exceptions, the majority of the isolates clustered together based on where they were isolated from. There are three main clusters: the first group G1 involves only four isolates, two from urine and two from blood. In G2, there are 20 isolates in three subclusters: the majority (17 isolates) were from the blood samples, with three exceptions (H28E, H32F and H30R). G3 contains 11 isolates from different sources, with the majority (6 isolates) from urine.

Phenotypic and genotypic markers, i.e., the 18 antibiotic resistance phenotypes, the 4 β-lactamase genes tested, the *Intl1* gene and the results for the phenotypic screening of ESBL, were also clustered (labelled on the horizontal axis). Four main clusters were identified, the most interesting of which are C3 and C4. In C3, nine different antibiograms clustered together with CTX-M carriage and the ESBL-screening composite measure: the quinolone members, including ciprofloxacin, enrofloxacin and nalidixic acid, clustered with ceftazidime and sulphonamide, and there was obvious clustering of cefotaxime with *bla*_CTX-M_, the ESBL-screening test, cefquinome, ceftiofur and aztreonam. In C4, *Intl1* and *bla*_OXA-1_ clustered with sulfamethoxazole–trimethoprim.

### 2.6. Phenotypic–Genotypic Association Using Chi-Squared Tests

**Figure 4:**
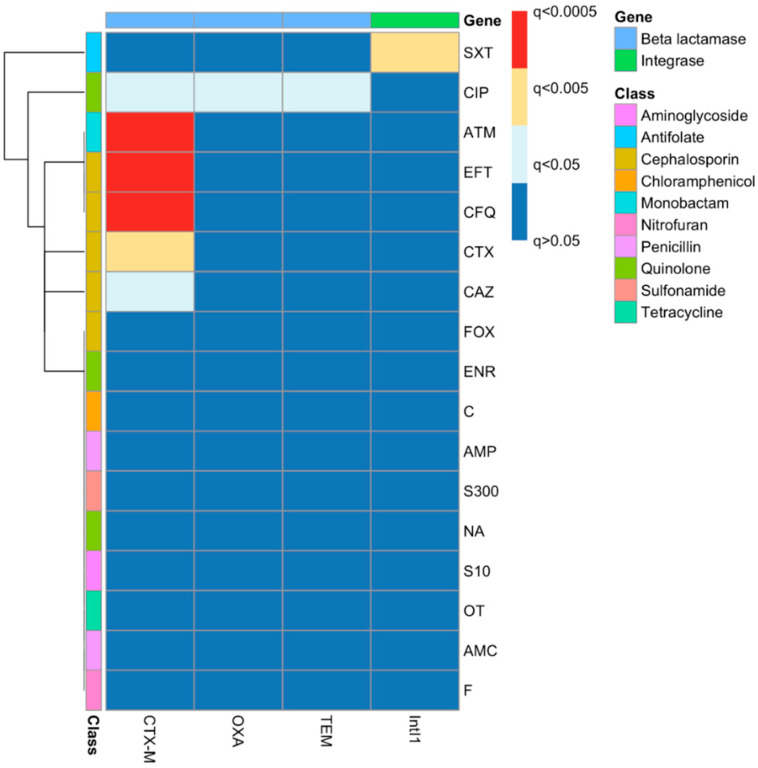
Associations between resistance genes and phenotypes as identified by chi-squared tests with false discovery rate corrections. The left side of the figure represents the different antibiotic resistance phenotypes that belong to different antibiotic classes, as clarified in the colour key, while the top of the figure represents the different genes that were tested.

The outcomes of chi-squared tests of associations between gene carriage and phenotypes were also clustered to aid in visualisation (Figure 4). There were significant associations between *bla*_CTX-M_ carriage and resistance to CTX, CAZ, EFT and CFQ, as well as to ATM and CIP. There was a significant association of the carriage of *bla*_OXA_ and *bla*_TEM_ only with ciprofloxacin and not with any of the beta-lactam antibiotics. The only significant association between *IntI1* carriage and the resistance phenotype is with trimethoprim–sulfamethoxazole resistance.

### 2.7. Genotyping of E. coli Strains Using ERIC-PCR

**Figure 5:**
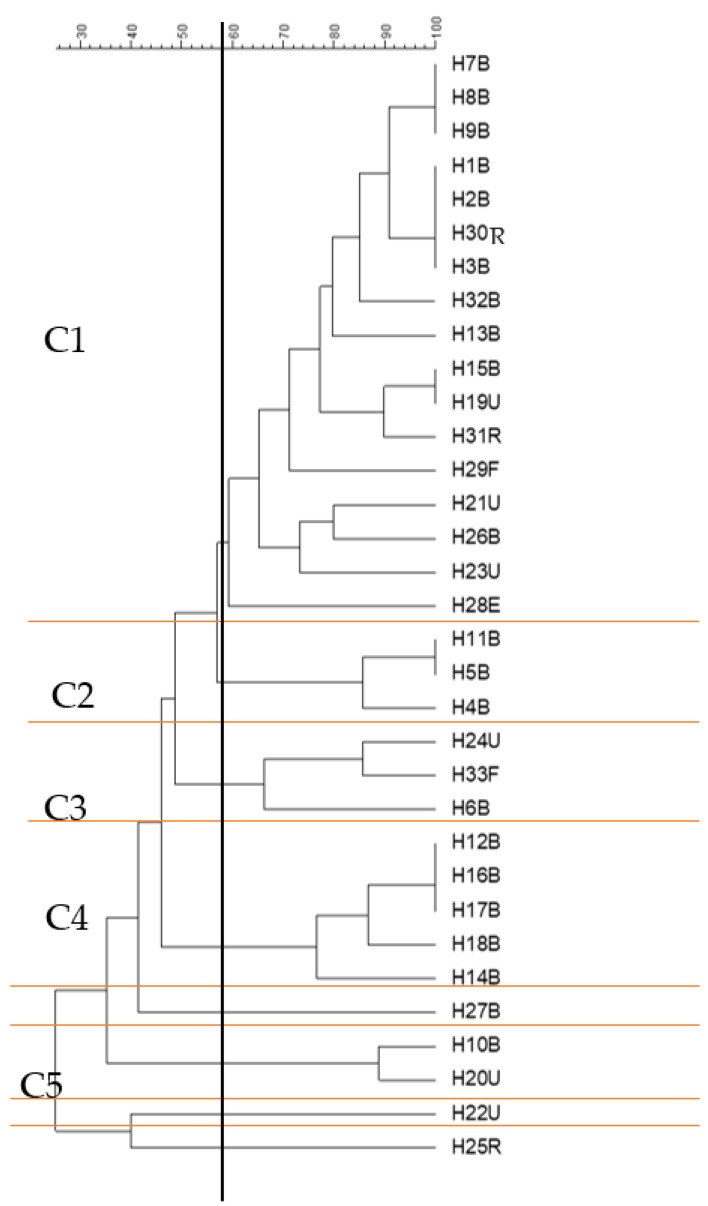
ERIC-PCR genotyping of 33 *E. coli* isolates from different clinical samples (clusters shown at 58% similarity level). The dendrogram was produced using the UPGMA algorithm based on the Dice similarity coefficient. H for human; B, U, F, R and E are specific designations to show the source of the sample. For example, B refers to blood, U is for samples collected from urine, F is for the sample collected from faeces; R is for samples collected from sputum from the respiratory system; and E is for the isolate collected from an ear infection. C refers to the cluster number. Clusters C1-C5 were formed at a 58% similarity level, as designated using AMOVA analysis. The horizontal black line divides each main group (C1-C5) that clustered at the 58% similarity threshold, which is indicated by the vertical black line.

The similarity of the ERIC-PCR profiles of the isolates from different samples was analysed, and cluster analysis divided strains into five main groups at a 58% similarity threshold, which cluster significance analysis demonstrated were mostly nonoverlapping and hence genomically independent groups. The cluster analysis divided isolates into five main groups (Figure 5), group 1 (n = 17), group 2 (n = 3), group 3 (n = 3), group 4 (n = 5) and group 5 (n = 2), with 3 outlier isolates, H27B, H22U and H25R, which did not cluster with any other groups at a 58% similarity threshold. This threshold for group delineation was based on the cluster significance analysis (ΦPT = 0.48; *p* < 0.001), as AMOVA statistical analysis using a higher % similarity threshold showed no significant difference amongst any of the branches. At 58% similarity, group 1 was significantly different from all other groups. This group was composed of 17 isolates (about 50% of the total number of isolates), which had been isolated from different samples but mainly from blood (11 isolates). All members of this group (except H29F and H23U) showed resistance to at least 10 of the tested antibiotics, with some of them resistant to 12, 13 and even 15 antibiotics. Remarkably, 11 isolates were carrying the *Intl1* gene, of which 8 were found in combination with *bla*_CTX-M_ and *bla*_OXA-1_ (the members of this group are printed in red typeface in Table 1). In the C1 group, some strains showed identical ERIC profiles, such as H7B, H8B and H9B; these also showed similarity in their gene carriage, as all carried *bla*_CTX-M_, *bla*_OXA-1_ and *Intl1*; however, there are slight differences in their antibiotic resistance profiles (Table 1). Similarly, H15B and H19U shared the same ERIC profile, but H15B was carrying *bla*_CTX-M_, *bla*_OXA-1_ and *Intl1*, while H19U was only carrying *Intl1*, and there was a slight difference in their antibiotic resistance profiles (Table 1).

Group 4 (C4) included five isolates, which were all isolated from blood infections. This group also included some isolates with similar ERIC profiles, which are H12B, H16B and H17B. H12B showed resistance to 15 antibiotics and was carrying *bla*_CTX-M_, while H16B showed resistance to 12 antibiotics and was carrying *bla*_CTX-M_ and *Intl1*, and H17B was resistant to 14 antibiotics but was not found to carry any of the tested genes (Table 1). Group 5, which was composed of two isolates, overlapped with the other groups (2, 3 and 4) and did not show a significant difference from them (Figure 5).

## 3. Discussion

Antimicrobial resistance surveillance is important for monitoring community infections in order to know about the resistance impacts of treatment strategies. *E. coli* is one of the groups of eight bacterial species that the WHO has highlighted as being of key AMR concern and serves as a sentinel organism for antimicrobial resistance development in blood and urine [37]. Resistance against β-lactam, cephalosporin and carbapenem antibiotics is increasingly documented, and many studies have reported increases in *E. coli* resistance to more than one class of antibiotics [38,39,40].

The *E. coli* isolates collected in this study were mainly from bloodstream infections (21/35), followed by isolates from urine samples (8). In England, *E. coli* represents the most common cause of bloodstream infections. In 2016, the incidence was 73.0 per 100,000 population, increasing to 77.7 per 100,000 population in 2019 [4]. In Nottingham, there was an apparent increase in bacteraemia infections caused by *E. coli* between 2012 (772 cases) and 2021 (831 cases) [13].

The isolates in this study were from clinical samples and were preliminarily identified as *E. coli,* but for confirmation, all the isolates were subcultured onto TBX agar, and then oxidase and indole tests were performed. Out of 35, 33 isolates were reconfirmed as *E. coli* due to their colony characteristics (green–blue colonies on TBX) and negative oxidase and positive indole tests [41,42]. Two of the isolates did not show the typical characteristics of *E. coli* on TBX, as they appeared as white colonies and were negative in oxidase and indole tests. When the 16S rDNA sequence was used to identify H10B, the blast results showed that it is 100% similar to *Klebsiella pneumoniae*.

The antibiotic resistance patterns of all of the isolates in this study were determined against 18 different antibiotics, with a focus on ESBL-mediated resistance. The antibiotics that were selected in this study belong to different classes of antibiotics that are used for different infection treatments in both humans and animals. Ceftiofur (third-generation cephalosporin), cefquinome (fourth-generation cephalosporin) and enrofloxacin (fluoroquinolone) are used exclusively for animal treatment [43,44,45,46]. Including antibiotics that are used for animal treatment allows the determination of the resistance pattern and their relation to other antibiotics belonging to the same antibiotic classes but are used only for human treatment. For example, ceftiofur and cefotaxime are both third-generation cephalosporins, but the first is used only for animal treatment, while the other is used for human treatment. Resistance to either ceftiofur or cefotaxime can arise from the presence of *bla*_CTX-M_, and understanding such examples of cross-resistance is important for antibiotic stewardship in the One Health approach.

In this study, the selection of isolates based on their resistance to one of the cephalosporins (for most of the isolates) revealed that most of those isolates were MDR *E. coli,* which were present in all clinical samples that we analysed. With regard to the level of resistance against the β-lactam antibiotic group, the isolates show high percentages of resistance against different antibiotics. Nine of the tested antibiotics belonged to the β-lactam group (Table 2). The highest percentage of resistance against penicillin was found in 97.1% of the strains. This was expected, as penicillin is the oldest generation of β-lactam antibiotics, and reported cases of resistance to penicillin are increasing worldwide. This is in agreement with another study in which 17 and 58 ESBL-producing *E. coli* isolates from UTI and blood were found to be resistant to ampicillin [47]. There was a variation in the level of resistance to the other members of the β-lactam group; cefotaxime and ceftazidime both belong to the third-generation cephalosporins, but the resistance to cefotaxime (71.4%) was higher than that to ceftazidime (54.2%). This might be due to the fact that the CTX-M enzyme usually has more hydrolytic activity against cefotaxime than against ceftazidime [48]. It is worth mentioning that the resistance level to ceftiofur (71.4%), which is a third-generation cephalosporin but used exclusively for veterinary purposes, was similar to resistance against cefotaxime, as all isolates that showed resistance to cefotaxime also showed resistance to ceftiofur. This supports previous findings that the development of resistance to one antibiotic could give cross-resistance to another in a chemically analogous antibiotic group, even if the antibiotic has had little or no use in the same host [49,50,51,52]. Although cefquinome is one of the fourth-generation cephalosporins and used for veterinary purposes, it did not show good effectiveness against any of the strains, as only 31.4% of the strains were sensitive. Cefoxitin (a second-generation cephalosporin that has a 7-α-methoxy group) in comparison showed more effectiveness compared to the other cephalosporins, as only 25.7% were found to be phenotypically resistant to this antibiotic. Resistance to cefoxitin is usually an indication of AmpC activity and not ESBL production, but when full phenotypic confirmation was performed for the nine isolates that showed resistance to cefoxitin, only one isolate was found to be phenotypically AmpC-positive, but without pAmpC detection, which might indicate that this phenotype is due to the overexpression of chromosomal AmpC. With regard to the resistance level for aztreonam, the isolates showed high levels of resistance (65.7%). No isolates showed resistance to imipenem, except for two isolates that showed intermediate resistance. This low level of resistance is similar to that from another study in China [40], where, of 100 *E. coli* strains isolated from different clinical samples, there were no isolates resistant to imipenem. In another study in Northeast India, the resistance levels to carbapenem were higher than in our findings, where, out of 62 *E. coli* isolates, 4 (6.4%) isolates were found to be carbapenem-resistant [53]. This variation in the level of resistance might be due to different treatment regimes followed in different hospitals and countries.

With regard to resistance to fluoroquinolone (ciprofloxacin and norfloxacin) and quinolone (nalidixic acid) antibiotics, there was a high level of resistance shown by the isolates. The similar resistance value (60%) for ciprofloxacin, which is a fluoroquinolone used for human treatment, and enrofloxacin, which is a fluoroquinolone used for veterinary purposes, indicates cross-resistance that occurs even if the antibiotic is not being used for human treatment. This is not surprising, as in one of our previous studies on *E. coli* isolates from a dairy farm, we found that all of the isolates that were resistant to enrofloxacin were also resistant to ciprofloxacin, even though ciprofloxacin had not been used on the farm [42].

Of the isolates, 57.1% were resistant to oxytetracycline; this antibiotic belongs to the tetracycline group of antibiotics and is a broad-spectrum antibiotic, active against a wide variety of bacteria [43]. The prevalence of resistance to this antibiotic class has steadily increased in clinical isolates from humans, increasing by 0.45% per year from 1950 to 2001 [38]. In the same study, it was found that resistance to sulphonamide had increased by 0.49% per year amongst human-isolated *E. coli*. Sulphonamide-resistant isolates were high amongst the isolates in the current study, as 74.2% of the isolates were resistant. This is higher than the results shown by another study [40], as only 47% of the isolates showed resistance to sulphonamide, and this might be due to differences between treatment regimes.

Chloramphenicol and nitrofurantoin showed higher effectiveness against the isolates, as 65.7% and 80% of the isolates showed sensitivity to chloramphenicol and nitrofurantoin, respectively. This shows some agreement with a previous study [54], as 85% of their *E. coli* isolates that had been isolated from neonatal UTI samples showed susceptibility to nitrofurantoin and chloramphenicol.

The CTX-M, TEM and SHV families represent the most frequently encountered ESBL genes in Enterobacteriaceae [31,55]. Of the 35 isolates in the current study, 24 were phenotypically confirmed as ESBL producers, and this was confirmed genotypically by detecting different β-lactamase genes. This study showed the presence of *bla*_CTX-M_ in 23/35 (65.7%) isolates, and it is worth mentioning that 24 isolates were detected as phenotypic ESBL producers, which indicates the effectiveness of the phenotype test that was performed. *bla*_TEM_ was detected in 37.1% of the isolates, and the most common subtypes among the definite sequence types were TEM-1 (75 %) and CTX-M-15 (40%). While *bla*_OXA-1_ was present in 34.2% of the isolates, it is notable that all isolates that carried *bla*_OXA-1_ carried *bla*_CTX-M_ as well, which might indicate the carriage of those genes on the same genetic elements. In a recent study using whole-genome sequencing, it was found that *bla*_OXA-1_-*bla*_CTX-M-55_-*tet(B)-aac(6′)-Ib-cr-dfrA17-sul1-fosA3* were all located on a *bla*_NDM-5_-harbouring self-transmissible IncX3 plasmid and a multireplicon IncFII/FIA/FIB plasmid [56]. Thus, the presence of specific resistance may be influenced not only by selection through antibiotic treatments but also by its genetic mechanism of carriage, as the selection of genes on a multiresistance replicon could occur through the use of only one of the antibiotics to which it provides resistance.

In a study on ESBL-producing *E. coli* and *K. pneumoniae* isolates from patients in a Northern Portuguese hospital, all of the *E. coli* isolates (38) that initially showed resistance to CTX/CAZ were ESBL producers. *bla*_CTX-M_ was detected in 37 isolates, and most of them (32 isolates) were *bla*_CTX-M-15_ [57], which is in agreement with the results of our study.

The *bla*_TEM_ variants detected in this study were mostly *bla*_TEM-1_ (7/13), with 2 of the isolates in this study (H5B and H26B) harbouring *bla*_CTX-M-15_ and *bla*_TEM-1_ together, while another isolate (H22U) was carrying *bla*_CTX-M-14_ and *bla*_TEM-1_. The co-carriage of *bla*_CTX-M-14_ and *bla*_TEM-1_ and *bla*_CTX-M-15_ and *bla*_TEM-1_ has been recorded before [23,58,59]. TEM-1 is a broad-spectrum beta-lactamase but is not an ESBL; it confers resistance to penicillin and early cephalosporins by hydrolysing their β-lactam rings. However, due to mutations in *bla*_TEM-1_, the active site plasticity increases due to the loss of hydrogen bonds, making the TEM more reactive to β-lactams [60]. It can be overexpressed, and it leads to cephamycin and carbapenem resistance [61].

*bla*_SHV_ was only detected in two isolates (H10B and H20U), which were confirmed as not *E. coli*. H10B was identified as *K. pneumoniae* and was also carrying *bla*_CTX-M_, *bla*_TEM_, *bla*_OXA-1_, *Intl1* and pAmpC, which belong to the CIT and FOX families, while H20U carried *bla*_SHV_, *bla*_TEM_, *Intl1*, *merA* and pAmpC, which also belong to the CIT and FOX families. *K. pneumoniae* carrying *bla*_SHV_, *bla*_CTX-M_ and *bla*_TEM_ has been seen before [59,62]. There is a strong possibility that H20U is also *Klebsiella pneumoniae*, as phenotypically, it showed the same characteristics as H10B, and it clustered together with H10B based on their antibiotic resistance profiles on the heatmap (Figure 3), and genotypically (using ERIC fingerprinting), it clustered with H10B in a separate cluster (C5) with a 91% similarity level (Figure 5).

In this study, *merA*, *merC* and *Intl1* genes were used to look for Tn*21*-like transposable elements, a mobile genetic element associated with mercury resistance and carrying a class 1 integron. This transposon (Tn*21*) and related transposons (Tn*1696*) frequently carry a mercury resistance (*mer*) operon, and usually, Tn*21*-like transposable elements carry a class 1 integron. Most resistance integrons belong to the class 1 integron family [63]. This integron category carries an integrase (*Intl1*), which is an essential component of an integron, and many class 1 integrons carrying antibiotic resistance cassettes are found on Tn*21*-like transposons (usually encoding resistance to streptomycin and sulphonamide), so this transposon is important in the movement of antibiotic resistance genes [26,28,64]. In this study, 17 isolates were found to be carrying *Intl1. merA* was detected in three isolates, and *merC* was identified in two isolates. However, only one isolate (H26B) was found to carry the three genes together (Table 1), which might indicate that a Tn*21*-like transposon is likely to be present in this isolate. It is worth mentioning that H26B is phenotypically resistant to streptomycin and sulphonamide, which gives more supporting evidence for this.

The resistance profiles of the majority of the isolates clustered together based on the source of isolation with some exceptions (Figure 3). The heatmap started from the top (G1) with MDR isolates down to the middle (G2) with an increasing amount of resistance (H12B–H11B). The bottom of the heatmap (G3) included the most sensitive isolates, such as H29F. The analysis revealed that, with some exceptions, the isolates that had been isolated from blood infections were more antibiotic-resistant compared to the other isolates, as is clear from the clustering of the isolates on the left-hand side (G2). The clustering of the phenotypes and gene carriage showed that there was a strong correlation between *Intl1* and *bla*_OXA-1_ clustered with SXT, which might indicate the presence of *bla*_OXA-1_ on a class 1 integron with another gene cassette that confers resistance to SXT (one of the *dfr* genes), as *bla*_OXA-1_*-dfr* has been reported before [65,66]. A chi-squared test showed there was a significant correlation between *Intl1* and the SXT resistance phenotype. Clinically, the most important mechanism for trimethoprim resistance is plasmid-mediated DHFRs [67]. As mentioned above, a class 1 integron may carry different antibiotic resistance gene cassettes, and as 11 isolates that carried *bla*_OXA-1_ were also carrying *Intl1*, this gene cassette might be carried by a class 1 integron. Looking at the genotypic cluster of cluster C1 in the ERIC dendrogram (Figure 5), we can see that C1 includes 11 isolates carrying *Intl1*, of which 8 are found in combination with *bla*_OXA-1_ and *bla*_CTX-M_. *bla*_CTX-M_ and the phenotypic ESBL-screening test were clearly clustered with cefotaxime, cefquinome, ceftiofur and aztreonam resistance, which indicates that the ESBL phenotypic testing carried out was a good indicator of the carriage of *bla*_CTX-M_. This was tested statistically using the chi-squared test, and it shows a significant correlation between *bla*_CTX-M_ carriage and resistance to CTX, CAZ, EFT and CFQ, as well as to ATM.

In summary, the phenotypic resistance pattern present in *E. coli* from different clinical samples was investigated, especially resistance to the β-lactam class of antibiotics. Different ESBL/pAmpC genes and their variant types were detected, and correlations between the phenotype and genotype were tested, together with the investigation of the presence of ESBL genes, Tn*21*-like transposons and class 1 integrons. Very high levels of resistance among ESBL-producing *E. coli* were observed, particularly among bloodstream isolates; those MDR ESBL-producing isolates cause serious human infections, thus making their treatment much more problematic. The continuous surveillance of AMR pathogens is a crucial aim to observe and manage the local situation and apply alternative therapeutic guidelines that can decrease the level of antibiotic resistance as much as possible.

## 4. Materials and Methods

### 4.1. Strains

Thirty-five putative *E. coli* clinical isolates were collected from Queen’s Medical Centre, Nottingham University Hospitals NHS Trust, Nottingham, UK. With the exception of urine isolates, the selection of the isolates was based on the demonstration of resistance to one of the cephalosporin groups of antibiotics. The isolates from urine were originally identified using MAST URI^®^*SYSTEM* (MAST URI^®^SYSTEM (mast-group.com, accessed on 1 September 2022)), while all other isolates were originally identified using Masterscan ELITE (MAST, Bootle, UK). All collected isolates were immediately stored as Microbank (Pro-Lab Diagnostics, Birkenhead, UK) bead stocks at –80 °C and were grown from frozen stocks for each subsequent characterisation. Further species confirmation was performed by culturing the isolates on tryptone bile X-glucuronide agar (TBX agar; Merck Cat. no. 1.16122) with overnight incubation at 37 °C; this culture medium is used as a selective medium to isolate and detect *E. coli*. TBX contains X-glucuronide, which is a substrate for glucuronidase carried by *E. coli* but no other coliforms. *E. coli* cells can take up colourless intact X-glucuronide. Intracellular glucuronidase cleaves the glucuronide–chromophore bond, releasing the coloured chromophore, which accumulates in cells, turning an *E. coli* colony blue/green [68]. All isolates that appeared blue–green on TBX were subjected to oxidase and indole tests as further confirmation [41]. The oxidase test was performed by using an oxidase strip (Oxidase Detection sticks, Oxoid Cat. no. MB0266). Several colonies were transferred to the diagnostic strip using a sterile plastic loop, and the results were read within 5–10 s. If no colour change was observed, it indicated a negative result, while the formation of a dark-blue colour was viewed as a positive result. For the indole test, tryptone water (Oxoid, UK catalogue number CM0087) was inoculated with one single colony of the tested bacteria, and the tubes were then incubated for about 24 h at 37 °C. In the second step, 0.2 mL of Kovacs’ reagent (Pro-lab Diagnostic, UK; Cat no. PL.375) was added to each tube and gently shaken. The development of a deep-red ring on the top of the broth indicated a positive result, while the negative reaction appeared as a light-yellow ring on the top of the broth [69]. Oxidase-negative and indole-positive strains were considered *E. coli*. *E. coli* ATCC 25922 (COSMOS BIOMEDICAL LTD, Swadlincote, UK) was used as a quality control strain for these tests.

The *E. coli* strains were isolated from different clinical samples and were designated to show the source and site of infection. For example, for isolate H1B, H is for human, 1 is the number of the isolate and B refers to blood. U designates isolates collected from urine, F indicates isolates collected from faeces, R represents isolates collected from sputum from the respiratory system, and E designates isolates collected from ear infections.

### 4.2. Antibiotic Sensitivity Tests

Antimicrobial sensitivity tests were carried out using the disc diffusion method according to Clinical & Laboratory Standards Institute (CLSI) and the National Committee for Clinical Laboratory Standards (NCCLS) guidelines with some modifications [69]. The test was carried out using 18 antimicrobials from seven different antimicrobial classes (Table 2). The inoculum for the test was prepared using a direct colony suspension method; approximately 2–4 colonies were taken from an 18 h incubated Luria–Bertani Lennox (LB) Agar (Fisher Scientific, Loughborough, UK) plate and were suspended in 5 mL of Mueller–Hinton broth (Oxoid, Basingstoke, UK). The bacterial suspension was then incubated for 2–6 h at 37 °C. The suspension volume was adjusted to achieve a turbidity equivalent to a 0.5 McFarland standard (Oxoid, UK), which results in a suspension containing approximately 1 to 2 × 10^8^ CFU ml^−1^. After dilution, a sterile swab was used to spread 100 μL of the culture over the surface of a 90 mm Petri dish that contained 25 mL of Mueller–Hinton agar (Oxoid, UK). The plates were left to dry at room temperature (for no more than 15 min), and sterile forceps or a disc dispenser (Oxoid, UK) was used to place the antimicrobial discs (Oxoid, UK) onto the plate surface. For each isolate, 4 plates of Mueller–Hinton agar were used. The plates were then incubated for 18–20 h at 37 °C, and the results were recorded by measuring the inhibition zone diameter across the disc; the zone of clearing was interpreted according to standard measurements [32,70]. To check the antimicrobial activity of the discs, the control strain *E. coli* ATCC 29522 was used as a negative control, which is sensitive to all antimicrobials used in the test.

### 4.3. Phenotypic Confirmation of ESBL/AmpC-Producing E. coli

The Total ESBL Confirm Kit (Rosco Diagnostica, France. Cat. no. 98014) was used to test ESBL production by the bacterial strains according to the guidelines of the manufacturer. Any bacterial isolates that produced an inhibition zone indicating resistance or intermediate resistance to cefotaxime and/or ceftazidime using standard antibiotic discs were further tested using a combination of discs containing cefotaxime (CTX), ceftazidime (CAZ) and cefepime (FEP) alone and in combination with clavulanate. Cefepime is not inactivated by chromosomally encoded AmpC to the same extent as other cephalosporins. The ESBL confirmation test was performed as described above for the antibiotic sensitivity test method. The positive presence of ESBL was recorded if there was an increase in the diameter of the zone of clearing around the disc by ≥5 mm for either antimicrobial agent tested in combination with clavulanate vs. the diameter of the zone of clearing around a disc containing the agent when tested alone [70]. The bacterial reference strains used were *E. coli* NCTC 13353 (ESBL positive control CTX-M-15, Public Health England, UK) and *E. coli* ATCC 25922 (ESBL negative control).

For the detection of de-repressed/plasmid-mediated AmpC β-lactamases, the AmpC Confirm Kit (Rosco Diagnostica, France. Cat. no. 98007) was used. The disc assay was performed as described above for the antibiotic disc assays. The test was performed for any isolates that showed resistance or intermediate resistance to cefoxitin (FOX), as it might indicate the overexpression of chromosomal AmpC. The results of the test were recorded as positive for any isolates that showed a ≥5 mm increase in the inhibition zone diameter of cefotaxime or ceftazidime in combination with cloxacillin, compared with the inhibition zone for the antibiotics alone, and indicated the presence of de-repressed/plasmid-encoded AmpC. *Enterobacter cloacae* NCTC 13406 (AmpC β-lactamase de-repressed, Public Health England, UK) was used as a positive control strain, while *E. coli* ATCC 29522 was used as a negative control strain.

### 4.4. Genotyping of Isolates

#### 4.4.1. DNA Extraction

Bacterial DNA for PCR was extracted using a simple boiling method [42]. (A single colony of *E. coli* was picked from an overnight culture on LB agar and placed in 100 μL of 1 × TE buffer (10 mM Tris-Cl, 1 mM EDTA buffer, pH 7.9.)) To disrupt bacterial cells, the suspension was heated to 99 °C for 30 min (Eppendorf Thermomixer comfort, Germany), and the sample was then centrifuged (Sigma1-16 bench-top centrifuge, UK) at 13,000× *g* for 15 min. The crude DNA in the supernatants was transferred into sterile microcentrifuge tubes and stored at −20 °C until required. The NanoDrop^®^ ND-1000 Spectrophotometer (Thermofisher, Loughborough, UK) was used to check the DNA concentration and purity of each sample. The ratio of absorbance at 260/280 nm was used to assess the purity of DNA, and a ratio of ~1.8–2 was generally accepted.

#### 4.4.2. ERIC-PCR

For genotype investigation, ERIC-PCR was used [71]. The PCR mixture (25 μL) contained 12.5 μL of DreamTaq Green PCR master mix (ThermoFisher Scientific, UK), 9.5 μL of nuclease-free water, 1 μL of DNA (50–100 ng) and 1 μL (10 pmol μL^−1^) of each primer (ERIC1 (forward) and ERIC2 (reverse)) (Eurofins MWG Operon, Germany) using the following conditions: 1 cycle of 3 min at 94 °C; 35 cycles of 30 s at 94 °C, 1 min at 52 °C, and 4 min at 65 °C. The final cycle was for 8 min at 65 °C. The PCR product (7 μL) was loaded onto a 2% (*w*/*v*) TAE (GelPilot^®^ LE Agarose, Qiagen, Hilden, Germany) agarose gel and electrophoresed at 120 V for 2 h. For cross-gel comparison, a 1 kb plus DNA ladder (Invitrogen, Waltham, MA, USA) was used. A Gel-Doc XR system (Bio-Rad, UK) was used to image all of the gels. Images were analysed using FPQuest gel analysis software (Bio-Rad, Hercules, CA, USA). A dendrogram was obtained from the comparison of ERIC-PCR profiles using the Dice coefficient and clustered by the unweighted pair group method with arithmetic averages (UPGMA) with 1.5% optimisation and 1.5% tolerance to display the dendrogram. To analyse the confidence of the selected similarity threshold and the significance of clusters, molecular variance framework (AMOVA) [72] was used. The AMOVA calculation was carried out using GenAlEx v6.5b5 software [73]. The significance was examined with the calculation of ΦPT, a measure of population differentiation that suppresses intraindividual variation. In the case of AMOVA, the null hypothesis (H0; ΦPT = 0) means that there is no genetic difference among the populations, and the alternative hypothesis (H1; ΦPT > 0) means there are genetic differences amongst the populations.

### 4.5. PCR Detection of Β-lactamase Genes

Β-Lactamase genes *bla*_OXA-1_ and *bla*_OXA-2_ and ESBL genes *bla*_SHV_, *bla*_TEM_ and *bla*_CTX-M_ were detected using PCR as described by Dierikx and co-workers [74]. All isolates that produced an inhibition zone indicating resistance or intermediate resistance to cefotaxime and/or ceftazidime using standard antibiotic discs were further tested for the presence of ESBL genes, regardless of the results of the phenotypic confirmation kit. The PCR reaction mixture (25 μL) included 12.5 μL of DreamTaq Green PCR master mix (2X) (ThermoFisher Scientific, UK), 8.5 μL of nuclease-free water, 1 μL (10 pmol μL^−1^) of each primer and total DNA (2 μL). The primers and expected product sizes are shown in Table 3. The reaction was carried out under the following conditions: one cycle of denaturation: 5 min at 94 °C, followed by 30 cycles of 30 s at 94 °C, 30 s at 55 °C and 60 s at 72 °C, with a final extension of 7 min at 72 °C. The PCR product (7 μL) was loaded onto a 1% (*w*/*v*) agarose gel (Melford Laboratories, UK) and electrophoresed at 80 V for 1 h. On each gel, a 100 bp plus DNA size ladder (Invitrogen, UK) was used. The PCR products for most of the positive isolates were sent for sequencing. *E. coli* NCTC 13353, *E. coli* NCTC 13352 and *Klebsiella pneumoniae* NCTC 13368 were used as positive quality control strains for the PCR reactions to detect *bla*_CTX_M,_ *bla*_TEM_ and *bla*_SHV,_ respectively_._ *E. coli* ATCC 25922 was used as a negative control for all PCR reactions.

### 4.6. PCR Detection of Plasmid ampC

The same isolates tested for ESBL genes were also tested for the presence of plasmid *ampC*, regardless of the results for the phenotypic confirmation kit. Primarily, tests were carried out using a set of primers to detect the CMY type of plasmid AmpC [75]; the PCR mixture (20 μL) contained 10 μL of HotStarTaq plus Mastermix (2X) (Qiagen, UK), 1 μL of each forward and reverse primer, 2 μL of CoralLoad dye (Qiagen, UK), 5 μL of DNase-free water and 1 μL of DNA. The PCR cycle consisted of an initial denaturing step at 95 °C for 5 min and then 30 cycles of 94 °C (30 s), 58 °C (30 s) and 72 °C (60 s). The PCR reaction ended with a final extension step at 72 °C for 10 min.

**Table 3:** PCR primers used for detection of beta-lactamase genes and ERIC-PCR primers.

Oligonucleotide Name	Sequence	Product Size (bp)	Reference
CTX-M-F	ATGTGCAGYACCAGTAARGTKATGGC	529 ^a^	[73]
CTX-M-R	TGGGTRAARTARGTSACCAGAAYSAGCGG		
TEM-F	GCGGAACCCCTATTTG	964	
TEM-R	ACCAATGCTTAATCAGTGAG		
SHV-F-	TTATCTCCCTGTTAGCCACC	796	
SHV-R-	GATTTGCTGATTTCGCTCGG		
OXA-1-F-	ATGAAAAACACAATACATATCAACTTCGC	820	
OXA-1-R-	GTGTGTTTAGAATGGTGATCGCATT		
OXA-2-F-	ACGATAGTTGTGGCAGACGAAC	601	
OXA-2-R-	ATYCTGTTTGGCGTATCRATATTC		
CMY-2-F-	ATGATGAAAAAATCGTTATGCTGC	1138	
CMY-2-R	GCTTTTCAAGAATGCGCCAGG		
MultiACC-F-	CACCTCCAGCGACTTGTTAC	346	[76]
MultiACC-R-	GTTAGCCAGCATCACGATCC		
MultiMOX-F-	GCAACAACGACAATCCATCCT	895	
MultiMOX-R-	GGGATAGGCGTAACTCTCCCAA		
MultiDHA-F-	TGATGGCACAGCAGGATATTC	997	
MultiDHA-R-	GCTTTGACTCTTTCGGTATTCG		
MultiCIT-F-	CGAAGAGGCAATGACCAGAC	538	
MultiCIT-R-	ACGGACAGGGTTAGGATAGY		
MultiEBC-F-	CGGTAAAGCCGATGTTGCG	683	
MultiEBC-R-	AGCCTAACCCCTGATACA		
MultiFOX-F-	CTACAGTGCGGGTGGTTT	162	
MultiFOX-R-	CTATTTGCGGCCAGGTGA		
ERIC-F-	ATGTAAGCTCCTGGGGATTCAC	variable	[70]
ERIC-R-	AAGTAAGTGACTGGGGTGAGCG		

^a^ (R is a purine; Y is a pyrimidine; S is G or C).

The PCR primers used in this study for the detection of β-lactamase and ESBL genes (CTX-M, TEM, SHV and OXA) and plasmid-mediated ampC (ACC, MOX, DHA, CIT, EBC, FOX and CMY-2) are shown. The correct PCR product sizes are shown for each primer pair. The ERIC-PCR primer sequences are also shown; PCR products from amplifications using ERIC are variable in size. All primers were supplied by Eurofins (MWG Operon, Germany).

Multiplex PCR was carried out as described in [75] to detect plasmid AmpC; the reaction targeted six families of *ampC* genes, namely, ACC, FOX, MOX, DHA, EBC and CIT. The PCR reactions were carried out in 50 μL of PCR mixture including 25 μL of DreamTaq Green PCR master mix (2X) (ThermoFisher Scientific, UK) and 1 μL of each reverse and forward primer for all except FOX and DHA, where 2.5 μL was used for each of the forward and reverse primers, with 5 μL of nuclease-free water and 2 μL of DNA. The PCR cycle started with an initial denaturing step at 94 °C for 10 min and then 30 cycles of 94 °C (40 s), 60 °C (40 s) and 72 °C (60 s). The PCR reaction ended with a final extension step at 72 °C for 7 min. All the primers are listed in Table 3.

### 4.7. 16S rDNA V3 Region PCR

Amplification of the V3 variable region of 16S rDNA was performed using a published protocol, which included the gene primer set: V3F (5′-CCTACGGGAGGCAGCAG-3′) and V3R (5′-ATTACCGCGGCTGCTGG-3′) [77]. The PCR mixture (25 μL) contained 12.5 μL of DreamTaq Green PCR master mix (2X) (ThermoFisher Scientific, UK), 9.5 μL of nuclease-free water, 1 μL of DNA and 1 μL (10 pmol μL^−1^) of each primer. The PCR cycle started with a denaturation step at 94 °C for 5 min. A touchdown PCR was then performed with an initial annealing temperature of 66 °C, which was decreased by 1 °C every cycle for 10 cycles; finally, the remaining 20 cycles were performed with an annealing temperature of 56 °C. The extension for each cycle was carried out at 72 °C for 3 min, while the final extension was at 72 °C for 10 min, then the PCR products (7 μL) were checked on 1.5% (*w*/*v*) TAE agarose gels and the PCR product was sent for sequencing.

### 4.8. Sequencing of PCR Products

The PCR products were sequenced by Eurofins (MWG Operon, Ebersberg, Germany) using Sanger cycle sequencing technology (dideoxy chain termination/cycle sequencing) on Applied Biosystems™ (ABI) 3730XL sequencing machines using ABI BigDye^®^ Terminator 3.1 chemistry. Sequences have been submitted to the NCBI databases, and the accession numbers are listed in Table 4.

### 4.9. Statistical Methods

Statistical analysis was carried out in R (version 4.2.0). Clustering of strains was carried out using the pheatmap library (version 1.0.12) with default clustering settings. Statistical associations between genes and phenotypes were carried out using chi-squared tests with q-values computed using Benjamini–Hochberg FDR [78], adjusted to account for multiple testing. Visualisation of the chi-squared test outcomes was also performed using pheatmap, with rows clustered with single-linkage clustering for aesthetic reasons.

## Figures and Tables

**Table 4:** Accession numbers for the sequenced genes.

Isolates ID	Gene	Variant Type	Accession Number
H1B	*bla*_CTX-M_	*bla*_CTX-M-55_	OP589230
H2B	*bla*_CTX-M_	*bla*_CTX-M-258_	OP679875
H3B	*bla*_CTX-M_	*bla*_CTX-M-15_	OP689692
H4B	*bla*_TEM_	*bla*_TEM-1_	OP703167
H5B	*bla*_CTX-M_/*bla*_TEM_	*bla*_CTX-M-15_/*bla*_TEM-1_	OP620950/OP703168
H8B	*bla*_CTX-M_	*bla*_CTX-M-254_	OP649440
H10B	16S rDNA	-	OP627530
H15B	*bla*_CTX-M_	*bla*_CTX-M-15_	OP620949
H20U	*bla*_TEM_	*bla*_TEM-1_	OP723109
H22U	*bla*_CTX-M_/*bla*_TEM_	*bla*_CTX-M-14_/*bla*_TEM-1_	OP679876/OP723108
H24U	*bla*_TEM_	*bla*_TEM-30_	OP703169
H25R	*bla*_TEM_	*bla*_TEM-32_	OP620947
H26B	*bla*_CTX-M_/*bla*_TEM_	*bla*_CTX-M-15_/*bla*_TEM-1_	OP689690/OP703166
H27B	*bla*_CTX-M_	*bla*_CTX-M-55_	OP689691
H32F	*bla*_CTX-M_	*bla*_CTX-M-245_	OP620948
H33F	*bla*_TEM_	*bla*_TEM-1_	OP649441

## Data Availability

The data presented in this study are all openly accessible in the article. The sequence types are openly available in GenBank, which is described in Section 4.
